# “Stop eating lollies and do lots of sports”: a prospective qualitative study of the development of children’s awareness of dietary restraint and exercise to lose weight

**DOI:** 10.1186/s12966-015-0318-x

**Published:** 2015-12-15

**Authors:** Rachel F. Rodgers, Eleanor H. Wertheim, Stephanie R. Damiano, Karen J. Gregg, Susan J. Paxton

**Affiliations:** Department of Applied Psychology, Northeastern University, Boston, USA; Laboratoire de Stress Traumatique, EA 4560, Université Paul Sabatier, Toulouse, France; School of Psychology and Public Health, La Trobe University, Melbourne, Australia

**Keywords:** Children, Dieting, Exercise, Prospective, Qualitative

## Abstract

**Background:**

Beliefs surrounding the usefulness of dietary restriction and physical activity as means of body shape and size modification is already present in children as young as 5-years-old, and these beliefs may increase the risk of unhealthy weight control behaviours later in life. To date, however, little is known regarding the development of these beliefs in younger children. The aim of the present study was, therefore, to explore young (aged 3- to 5-years old) children’s conceptualisations of dietary restriction and physical activity as means to change body size using a prospective approach.

**Methods:**

A sample of 259 children (116 boys, 143 girls) participated in interviews at 3-, 4- and 5-years-old. Participants were shown silhouette figures of a child of their gender and age. Their responses to questions regarding how the figure could return to a previous thinner shape were qualitatively coded using thematic analysis.

**Results:**

Children’s responses revealed that while, for a subsample, modifications of food, eating, and exercise patterns were the most salient ideas, a number of other mechanisms of body change were also suggested. Responses also evidenced adoption or awareness of stigmatising attitudes towards overweight individuals (over 15 % by age 5). The proportion of children demonstrating an awareness of dietary restriction and physical exercise as methods for body size change increased significantly at each time point. While only 4.2 % demonstrated dieting awareness at 3-years-old, this proportion had risen to almost 28 % by 5-years-old (*p* < .001). Similarly, the proportion of children aware of exercise as a body change strategy rose from 2.3 to 16.3 % (*p* < .001), with 22 % of 5-year-olds mentioning general physical activity as a strategy. No gender differences were found.

**Conclusions:**

Awareness of dietary restriction and physical exercise as strategies for weight loss and body change emerges as young as 3-years-old, and significantly increases from 3- to 5-years-old. Interventions aiming to promote healthy means of weight control and obesity prevention should consider that certain attitudes may already be present in very young children.

## Background

With the concurrent rise in rates of obesity and disordered eating [1], Western society is increasingly focused on weight control. The prevailing social discourse holds that the two main ways of achieving this are through dietary restriction and physical exercise, which are promoted as the keys to modifying shape and weight [2]. It has been argued that, while physical activity is protective of pediatric obesity [3], these strategies are relatively ineffective in sustainably modifying weight and shape in healthy ways [4, 5], and when taken to extremes they may lead to negative outcomes including depression, eating disorders, obesity and metabolic diseases, excessive exercise and physical complications [6–10]. Dieting in children is widespread with up to a third of 5-year-old children reporting engaging in dieting related behaviours [11, 12], and rates reaching one in ten among children aged 9 to 12 years old [13]. While a host of research has examined body change strategies in adolescents and adults, and some research has examined children, to date, very little is known regarding the development of awareness of weight control or weight loss behaviours in pre-school children. The present study aimed to explore young (aged 3- to 5-years-old) children’s conceptualisations of dietary restriction and physical activity as a means to change body size using a prospective approach.

A limited number of studies have examined awareness of restricting dietary intake and exercising as methods of weight-control in children as young as 5-years-old [14–17]. Two cross sectional studies explored 5-year-old girls’ understanding and awareness of dietary restriction [14, 16]. In response to what makes people thin, 35 % of children gave reasonable responses such as suggesting it involved modified eating behaviours (12 %), restricted eating behaviours (14 %), exercise (6 %), losing weight (4 %), or other reasonable responses (3 %) [16]. In response to what people do when they diet, as many as 55 % gave relevant responses. Thus, a substantial subgroup of children appear to already have a concept of dieting and dietary restriction to lose weight at 5-years-old with a smaller group focusing on exercise behaviours as a way to become thin.

Two longitudinal studies have investigated the development of awareness of dietary restriction and physical activity as body change strategies in 5- to 7-year-old children [15, 17]. These studies revealed that approximately half of the 5-year-old children interviewed could articulate some ideas around dietary restraint and exercise for weight and shape modification [14]. Furthermore, children’s awareness of the strategies appeared to increase between the ages of 5- and 7-years-old [15, 17].

To our knowledge no studies have investigated the development of awareness of dietary restriction and exercise as methods of shape and weight change in children younger than 5. This is an important gap as the extant research suggests that by age 5-years-old a substantial proportion of children have already developed an understanding of dietary restriction, exercise and weight and shape change strategies [16], and are engaging in dietary restriction [12, 18]. In addition, the studies described above were conducted a decade ago and it is important to understand how the increasing social discourse surrounding weight control affects very young children. Furthermore, children’s awareness of weight control strategies has been shown to be associated with low self-esteem, higher body mass index (BMI) and body dissatisfaction [19], all of which predict future dieting behaviours [20, 21]. Given that dietary restriction is a risk factor for disordered eating and overweight among youth [22, 23], understanding the development of children’s conceptualisations and awareness of body size changes and weight control strategies from an early age, even before the age of 5, is important and may inform early prevention and intervention programs.

The aim of the present study was, therefore, to investigate the prospective development of awareness of dietary restriction and physical exercise as body change strategies in very young girls and boys starting at the age of three. These questions are important since they shed light on the development of body image concerns surrounding weight and shape, and the age at which different types of weight control methods, including potentially risky ones, may start to manifest. Specifically, we hypothesised that as children grow older, the proportion of children demonstrating an awareness of weight loss strategies would increase, with more children demonstrating awareness of dietary restriction and/or exercise for weight control.

A second aim was to determine whether there were gender differences in this age group. Among older children and adolescents, gender differences are typically found, with girls being more likely than boys to report wanting to lose weight and to use dieting as a method to do so [24]. Contrary to this, however, the only study to have formally compared awareness of dietary restriction between boys and girls in 5- to 8-year-olds found no gender differences [15], suggesting that gender differences may emerge later in childhood. Therefore, it was expected that in our study, no gender differences would be found in initial rates of body change strategies from 3- to 5-years-old.

## Methods

### Participants

The sample included 259 children (116 boys, 143 girls) who had responded to interviews at 3-, 4- and 5-years-old. Participants from Melbourne, Australia were recruited in the *Children’s Body Image Development Study* when children were 3-years, by placing advertisements in childcare centres and playgroups. Of 361 families who expressed interest in the study, three declined to participate and 63 could not be contacted or moved before data collection started, yielding an initial sample of 295 children. Participants completed assessments at baseline (Time 1) and at two one-year intervals (Time 2 and Time 3). At Time 2, 16 children (5 %) had moved and therefore were no longer included in the study, resulting in 279 participants at Time 2. No other participants were lost between Time 1 and Time 2. At Time 3, 20 children (7.2 %) had moved or parents had new work commitments preventing continuing, resulting in 259 children at Time 3 with complete data at all three time points. Postcode data indicated that of the initial sample, most families (58 %) lived in high socioeconomic areas, 32 % lived in average areas, and 10 % in disadvantaged areas (Australian Bureau of Statistics, 2011). Most parents were well-educated (77.1 % of mothers and 65.4 % of fathers had completed a university degree).

### Measures

Demographic data on child age, postcode of the child’s residence, and education level of parents were obtained from the primary care giver (98 % mothers) in a self-report questionnaire.

#### Understandings of body size changes

Children’s understandings of body size changes and methods for decreasing body size were assessed using an adaptation of a child figure rating scale [25] and with a method previously used by Lowes and Tiggemann (2003), as described elsewhere [26]. At 3- and 4-years old, children were presented with two felt fabric silhouettes 20 cm in height, representing child figures matching the child’s gender. At 5-years-old the silhouettes were the same sizes, but laminated. The first figure was an average body size, corresponding to third figure of the scale previously used [26]; the other figure was overweight, corresponding to the largest figure. Children were told the figure was their age, and “There was a little boy/girl who used to look like this [interviewer pointing to thinner figure], and now he/she looks like this [pointing to larger figure]. So, he/she is the same height [demonstrating with hands at head and feet], but he/she has changed”. Children were asked how the child figure was different, and prompted to notice that the figure got bigger around the abdomen if they did not note that. They were asked two questions “What do you think this boy/girl should do?” and then, “If this boy/girl [larger figure] wanted to change back to look like this again [pointing to thinner figure] how could he/she do that? If there was no response, children were prompted with “What do you think this boy/girl could do to look like that [pointing to thinner figure]” or “Just take your best guess” or “Any ideas?”

#### Meaning of the word ‘diet’

At Time 3 only, the interviewer asked the child, “What do you think a diet might be? Any ideas?” This was followed by prompts to take their best guess.

### Procedures

The project received University Human Ethics Committee approval. Families were recruited into the prospective Children’s Body Image Development Study, when children were 3-years-old. Parental written consent for their own and their child’s involvement was obtained, and children provided verbal assent prior to the interview.

Interviews were conducted in the child’s home (with a parent present) by trained interviewers with experience in standardized psychological testing of young children. Interviewers were encouraging of all child responses and children were told “there are no right or wrong answers to my questions. I’m just here to find out what 3/4/5-year-old children think about things”. Children answered a series of questions including the child figure rating based questions reported here and quantitative measures reported elsewhere [26, 27]. At completion of each time point interview, children were given a sticker and families provided with an AU$10 shopping voucher and entered in a prize draw for further vouchers. Parents were recontacted at yearly intervals to complete measures at Time 2 and Time 3. Multiple strategies, such as personal contact to organize child interviews and regular communication through newsletters and Christmas cards, were employed to reduce participant attrition between time points.

### Data analyses

#### Inductive identification and coding of themes

A thematic analysis was conducted, using an inductive approach in which theme identification is driven by the data [28]. Thematic content analysis allows for the subjective interpretation of text data through the systematic classification process of coding and identifying themes or patterns [29, 30].

Children’s responses were transcribed and entered into a spreadsheet displaying each child’s responses to the figure-based questions at each time point. Data from all three time points were coded simultaneously with coders blind to the child’s gender and data from different time points intermingled. The coding team included two psychologists specialising in body image and disordered eating (authors 1 and 2), and one researcher in that field (author 3), who identified themes and sub-categories of responses within themes. These three raters, who independently identified very similar themes, then discussed similarities and discrepancies in theme categorisation and arrived at consensus on a series of major themes (e.g., “Diet/food strategies”) and sub-categories within each theme (e.g., “Eat less quantity” or “Change quality of food” – which were sub-categories of “Diet/food strategies”). Table 1 displays major themes and subcategories. Responses to the question regarding the meaning of ‘diet’ were also analysed for themes.

**Table 1:** Data-derived themes related to changing from larger to thinner figure with sample quotes

Themes	Sample quotes
Diet/food strategies	
Eat less quantity	“eat a little bit of food”, “don’t eat much”, “eat once a day”, “not eating so much food”
Don’t eat/stop eating	“don’t eat”, “stop eating food”, “she eats nothing”, “not eat for two days or one”
Change quality of food	“eat… proper food”, “good things, not sugar”, “carrots”, “veggies”, “stop eating lollies”
Eat “healthy” food	"eat healthy food/stuff”, “she would eat hundreds of healthy food”, “she has to eat healthy fruit”
Vague reference to food/eating	“cook food”, “just eat”
Physical activity	
Exercise	“do exercising ‘til he gets back to small”, “running”, “go to the gym”
Active play	“jumping”, “go to the park and play”
Other physiological strategies	
Drink water	“drink water – lots and lots”
Vomit or spit	“could vomit”, “get the food out”, “spit out all the food”
Medical/ scientific interventions	“go to the doctor’s”, “go to the hospital”, “ask a scientist to make him back to normal”
Go to toilet	“go on the toilet”, “do poohs”
Have baby/take baby out	“getting the baby out of her tummy”
Play (non-specific or sedentary)	“play”, “play outside”, “play with blocks”, “play video”
Other physical mechanisms	“burp”, “blow her breath out”, “go under water”
Negative implications of large size	
Ostracism, withdrawal	“he's too fat, people would laugh at him”, “play with fat people”, “move away from the other girls”, “go in jail”, “she should kill herself and then she’ll grow back again”
Disobedience	“be naughty – he’s lazy”, “shove people and push them”
Should lose weight, change back	“[should] change back to the smaller one”, “go back to the body before”
Negative/distressing implications	“sad and crying”, “I don’t want to change into that one…”, “fall over and smash people, he’s so fat”
Positive implications	
Getting stronger	“now he is very strong”, she’s healthier”
No need to change /accept body	“stop talking about how she looks”, “can still do everything”
Unrelated explanations	
Magical or imagining	“by magic”, “a fairy would come”, “time machine”, “wish and wish and wish”
Clothing responses	“put a belt on”, “take her clothes off”, “get different clothes on”
Unsure/no response	“don’t know”
Can't change	“he/she can’t [change back to the thinner figure]”, “it’s impossible”, “no way!”

*N* = 259

To determine frequency of responses in each of the thematic categories, two raters (this time authors 3 and 4) reviewed the age and gender non-identified data set, coding each response according to whether it represented each thematic category. Responses could be coded as belonging to more than one theme. A third rater (author 5, a senior author) reviewed all discrepant codings and made a final decision. To quantify responses, participants were allocated a score of ‘1’ for a theme if their answer to *either* the question related to what the child figure does next *or* how to reduce the figure body size was coded as representing that theme.

#### Theory driven coding of key awarenesses

A further a priori theory-driven approach was used to address key hypotheses regarding dietary restriction and physical exercise awareness (top of Table 2). First, a *diet awareness* score of ‘1’ was allocated if, on either of the questions (what the larger figure would do next, or as a method for the larger figure to become thinner), a participant’s reference to food involved modifying food intake through reducing or stopping eating or changing the quality of foods eaten. Second, a more general *food awareness* score of ‘1’ was allocated whenever a participant mentioned food, eating or cooking in response to either of the two questions, regardless of the quality of the answer. Thus, the *diet awareness* scoring represented a more restrictive subcategory of the *food awareness* score. An *exercise awareness* score of ‘1’ was allocated to participants who mentioned exercise or a type of physical activity likely to be associated with an exercise regime for weight loss, such as running. A second, more general, *physical activity awareness* score of ‘1’ was allocated if participants suggested either the exercise-related activities included in the category just described *or* active play, such as playing at the park or jumping. Thus, the *exercise awareness* scoring represented a more restrictive subcategory of the *physical activity awareness* score. For each of these four indices children whose responses did not indicate an awareness of the construct being coded received a score of ‘0’. Inter-rater reliability was high with rates of 91 % agreement for *diet awareness,* 95 % for *food awareness,* 86 % agreement for the presence of *exercise awareness*, and 82 % for *physical activity* awareness.

**Table 2:** Differences between time points (ages 3 to 5) in percentages of children responding according to each theme

Themes	Time 1^a^	Time 2	Time 3	Cochran’s Q	McNemar’s test		
	% (*n*)	% (*n*)	% (*n*)		T1 vs. T2	T1 vs. T3	T2 vs. T3
Primary theory-driven coding categories							
General food awareness^b^	6.2 (16)	18.1 (47)	29.3 (76)	Q(2) = 51.94, *p* < .001	*χ* ^2^ = 18.36***	*χ* ^2^ = 43.5***	*χ* ^2^ = 9.92**
Diet awareness^b^	4.2 (11)	15.4 (40)	27.8 (72)	Q(2) = 59.43, *p* < .001	*χ* ^2^ = 19.12***	*χ* ^2^ = 49.31***	*χ* ^2^ = 12.98***
Physical activity awareness^b^	6.6 (17)	16.2 (42)	22.0 (57)	Q(2) = 24.02, *p* < .001	*χ* ^2^ = 10.11***	*χ* ^2^ = 22.37***	*χ* ^2^ = 2.48, *ns*
Exercise awareness^b^	2.3 (6)	11.6 (30)	16.3 (50)	Q(2) = 59.42, *p* < .001	*χ* ^2^ = 14.69***	*χ* ^2^ = 34.24***	*χ* ^2^ = 5.31*
Data-derived themes							
Eat less	1.2 (3)	5.8 (15)	8.9 (23)	Q(2) = 15.59, *p* < .0005	*χ* ^2^ = 7.56**	*χ* ^2^ = 13.88***	*χ* ^2^ = 1.36, *ns*
Stop eating	0.8 (2)	5.4 (14)	4.6 (12)	Q(2) = 9.54, *p =* .008	*χ* ^2^ = 7.56**	*χ* ^2^ = 5.78*	*χ* ^2^ = 0.04, *ns*
Change quality of food	2.3 (6)	5.0 (13)	15.1 (39)	Q(2) = 43.19, *p <* .001	*χ* ^2^ = 3.27, *ns*	*χ* ^2^ = 26.25***	*χ* ^2^ = 18.39***
Change specific foods	1.5 (4)	3.5 (9)	8.1 (21)	Q(2) = 14.77, *p <* .001	*χ* ^2^ = 1.45, *ns*	*χ* ^2^ = 10.24***	*χ* ^2^ = 4.65*
Eat “healthy” food	0.8 (2)	1.9 (5)	8.9 (23)	Q(2) = 32.25, *p* < .0005	*χ* ^2^ = 0.80, *ns*	*χ* ^2^ = 19.05***	*χ* ^2^ = 13.14***
Vague food reference	1.5 (4)	2.7 (7)	1.2 (3)	Q(2) = 1.86, *p* = .247	--	--	--
Vomit or spit out	1.2 (3)	3.1 (8)	2.3 (6)	Q(2) = 3.75, *p* = .305	--	--	--
Drink water	0.8 (2)	0.8 (2)	1.2 (3)	Q(2) = 0.28, *p* = .870	--	--	--
Medical/scientific intervention	0.4 (1)	1.9 (5)	2.3 (6)	Q(2) = 3.82, *p* = .148	--	--	--
Negative implications	10.0 (26)	17.4 (45)	15.4 (40)	Q(2) = 6.47, *p* = .039			
Positive implications	1.9 (5)	3.1 (8)	2.3 (6)	Q(2) = 0.78, *p* = .68	--	--	--
Clothing-related	1.5 (4)	4.6 (12)	1.9 (4)	Q(2) = 5.70, *p* = .058	--	--	--
Aging-related	2.3 (6)	1.9 (5)	1.5 (4)	Q(2) = 0.40, *p* = .82	--	--	--
Have a baby	0.8 (2)	1.5 (4)	0.8 (2)	Q(2) = 0.00, *p* = 1.00	--	--	--
Go to toilet	1.2 (3)	1.9 (5)	2.3 (6)	Q(2) = 1.00, *p* = .607	--	--	--
Play (non-specific or sedentary)	9.7 (25)	18.5 (48)	10.8 (28)	Q(2) = 10.54, *p* = .005	*χ* ^2^ = 7.93**	*χ* ^2^ = 5.31*	*χ* ^2^ = 0.08^¶^
Magic/imagination	9.7 (25)	18.5 (48)	10.8 (28)	Q(2) = 1.94, *p* = .379	--	--	--
Can’t change	1.9 (5)	4.6 (12)	10.0 (26)	Q(2) = 16.73, *p* < .001	*χ* ^2^ = 2.12, *ns*	*χ* ^2^ = 12.90***	*χ* ^2^ = 4.97*
Miscellaneous/ambiguous	23 (68)	18.5 (48)	6.2 (16)	Q(2) =, 29.07, *p* < .001	*χ* ^2^ = 1.15, ns	*χ* ^2^ = 28.00 ***	*χ* ^2^ = 17.16 ***
No response	35.9 (93)	12.4 (32)	12.0 (31)	Q(2) = 66.37, *p* < .0005	*χ* ^2^ = 39.56***	*χ* ^2^ = 31.34***	*χ* ^2^ = 0.00, *ns*

^a^Time 1 = age 3, Time 2 = age 4, Time 3 = age 5; *N* = 259

^¶^ *p* < .10, * *p* < .05, ** *p* < .010, *** *p* < .001

^b^Diet awareness and exercise awareness were coded based on a priori theoretical grounds and are subsets of the broader categories general food awareness and physical activity awareness respectively

#### Analysing developmental and gender differences

Because of the dichotomous nature of the variables, nonparametric tests were conducted. Cochran’s Q tests were conducted to determine significant differences in the proportions of children mentioning each theme (versus those who provided a different response and those providing no response grouped together) across the three time points. When Cochran’s Q was significant, follow-up McNemar tests were conducted comparing pairs of time points. To examine gender differences, chi-square tests were conducted. The primary analyses focused on differences in diet awareness, food awareness, physical activity awareness and exercise awareness. Exploratory analyses examined differences for other individual coded themes; because of the multiple analyses conducted, results from these analyses were considered significant at an alpha level of .01.

## Results

Most children offered responses to at least one of the prompting questions, although 35, 12 and 12 % of 3-, 4- and 5-year-olds respectively did not respond, or indicated they did not know. At age 3, 44 % of children offered a response that received only one code, 53 % of children gave a response that received two codes, and 3 % of children gave a response that received three codes. At age 4, 44 % of responses received one code, 49 % of responses received two codes, 6.6 % of responses received three codes, and 0.4 % of responses received four codes. At age 5, 48.2 % of responses received one code, 45.6 % of responses received two codes, 5.8 % of responses received three codes, and 0.4 % of responses received four codes.

Table 1 displays inductively derived themes and subcategories identified from the children’s responses to questions related to what the larger size child figure should do or how it could turn back into the thinner figure. Representative quotes are included. Major themes included diet/food strategies, physical activity, other physiological strategies, unrelated explanations, can’t change, negative (and positive) implications of large body size, and unsure/no response. In the text that follows key sub-categories (sub-themes) are described.

### Qualitative responses relating to methods for reducing body size and weight

#### Eat less quantity or stop food intake

A range of responses indicated that children believed that reducing food intake would influence body size. These responses took forms such as eating less, including eating a small amount (1.2, 5.8, and 5 % at the three time points respectively) or suggesting stopping eating altogether (0.8, 5.4, and 4.6 %).

#### Change quality of food

Overall, responses suggesting eating particular quality foods were provided by 2.3, 5, and 15 % of children across the three time points. Responses sometimes were specific to types of food, and other times referred to their status as “healthy” foods. Some children (1.5, 3.5, and 8 % at each time point) suggested changing the quality of food eaten in specific ways. Foods to eat or eat more of included “fruits and vegetables”, “carrots”, “apple” and in one case “biscuits”. In other instances children suggested reducing “bad food”, “snacks”, “treats”, “sweets”, “lollies and chocolates” or, more rarely, “fat”. Another response (0.8, 1.9, and 8.9 % of children at the three time points respectively) involved making changes towards foods that were described as “healthy”. Only one child (a 5-year-old boy) used the term “diet”.

#### Physical activity

“Exercise” or specific forms of it such as running or going to the gym were suggested by 2.3, 11.6, and 16.3 % of children at each time point, and a further 2.7 to 3.8 % across the 3 time points suggested some form of active play, such as playing at the park or skipping.

#### Purging behaviours

A small minority of children at all ages suggested forms of purging. These included, “she could vomit”, “get the food out”, and “spit out all the food” (1.2, 3.1 and 2.3 % across time points). A small number of children made suggestions about evacuating bowels, such as “go on the toilet”.

#### Medical or scientific intervention

Some form of medical or scientific intervention was offered by 0.4, 1.9, and 2.3 % of children across the three time points, such as “go to the doctors”, or “ask a scientist to make him back to normal”.

#### Other physiological or physical strategies

Several children noted other physical or physiological mechanisms which suggested limited understanding of the cause of body size, such as burping or blowing breath out. A further theme involved changes in clothing, including taking off clothes or putting a belt on. Some children interpreted the larger figure as pregnant and suggested “get the baby out”. A small minority of children offered responses indicating aging, such as to stop having birthdays.

#### Playing

A substantial number of children (9.7, 18.5, and 10.8 %) suggested “play” of some sort. Active forms of play, such as “skipping and jumping” and “run around” were coded as part of the physical activity category. However, other forms of play mentioned were more sedentary or unrelated to exercise, such as playing with blocks or watching videos. In these instances, play appeared not so much a mechanism for size reduction, as just an enjoyable thing to do.

#### Magical intervention and wishful thinking

Story-like associations were evoked by 9.7, 18.5, and 10.8 % of children (across time points respectively). Children suggested that ways to reduce the size would be through magical intervention or through wishing or imagining. While these suggestions may have been due to children taking the assessment process as a story or game, and prompts to make a guess, it is also possible magical solutions were evoked because it seemed unlikely the figure could change to such an extent, reversing considerable growth, in reality.

#### “She can’t change”

Consistent with the latter suggestion, when asked how the child figure could change back to the thinner size, at the three time points 17.4, 4.6, and 10.0 % of children suggested it was not possible, with some children making vehement comments such as, “no way!”

### Qualitative responses associating body size change with negative or positive implications

While most children simply answered the questions asked regarding figure size changes, some children made comments implying that the change in body size had substantially negative implications, and a small minority of children (no more than 3 %) suggested positive implications.

#### Negative associations with body size: Body image stigmatisation and stereotyping

This theme, present in 10.0, 17.4 and 15.4 % of children across the three time points, involved suggestions that a large body size was negative and the source of stigmatising responses by others, as in “he’s too fat, people would laugh at him”. These comments sometimes took the form of disconnection from others, through being ostracised, having to play alone or playing exclusively with fat people. In one case (5-year-old), the response was more extreme, “she should kill herself and then she’ll grow back again”. Other responses suggested stereotyping involving the association of the larger figure size with negative personal characteristics, typically being disobedient, as in, “naughty”, “greedy”, “pushing others” or “lazy”.

#### Positive implications of body size

Positive implications were noted in 1.9, 3.1 and 2.3 % of children, as in “she’s healthier”, “now he is very strong”, and “he was always starving”. Some children noted that the change was desired, as in “wanted to be fat” or “she didn’t like the way she looked [when thinner]”. One 5-year-old girl appeared to have an anti-fat-talk body acceptance stance saying, “stop talking about how she looks”.

### Defining “Diet”

At Time 3, when children were 5 years, their understanding of the meaning of diet was explored. The vast majority (87.5 %) said they did not know or responded “what’s a diet?” A few children (6.5 %) offered responses suggesting a misunderstanding (“like you died?”) or an inaccurate (“hairstyle”) or vague attempt (“if it’s not good or bad for you”).

#### Associating diets with food

Twenty-one children (8.1 % of the sample) correctly linked “diet” with food. These definitions varied as to how articulate they were, ranging from simple associations with food (“it has food in it” or “yes, about food”) to 10 children (3.8 % of the sample) who provided descriptions that included the concept of food rules or dietary restriction. Examples of these latter definitions included “something that you’re supposed to eat and not supposed to eat”, “you only eat meat and fish and chicken”, “you have to eat healthy food” and “you don’t eat the foods that you are on a diet on”.

#### Associating diets with appearance

Four children (1.5 %) said their parent was on a diet, three of whom noted the diet involved food rules. Examples included: “My dad's on a diet. You can’t eat yuck food”, “My mum has a diet - you can't eat food” and “It’s when you stick to eat things - Mum is on a gluten free diet”.

#### Associating diets with appearance

Three children (1.2 %) identified dieting as a means of attaining a desired appearance. Responses included: “it’s when you change back to what you want to look like” and “it makes you all skinny again”.

### Developmental differences

Table 2 displays percentages of participants whose answers were coded as falling into each of the different themes. Differences between time points in percentages of children responding within each theme are displayed.

Cochrane Q tests for the four main a priori theoretically derived variables of *diet awareness* (themes: reduce/stop food intake and change quality of food), *food awareness* (all food themes, including vague reference), *exercise awareness*, and *physical activity awareness* showed significant differences between time points, *p* < .001. For all four main themes, the percentage of children indicating awareness increased with age. McNemar tests comparing each pair of time points indicated that between ages 3 and 4, and between 3 and 5 all four awareness variables revealed significant increases, *p* ≤ .001. Between ages 4 and 5, significant increases were found for food and diet awareness scores (*p* < .001) and physical activity awareness (*p* < .05), but not for exercise awareness. Exploratory analyses of inductively derived subcategories suggested that these differences were due to changes in the proportions of children referring to changes in the quantity or type of foods eaten. For both “eating less” and “changing the quality of food,” the proportions of children mentioning these at age 3 (less than 2 %), had significantly increased by age 4, increasing again to 8–9 % by age 5. The proportion of children recommending stopping eating altogether increased significantly from age 3 to 4, from 0.8 to 5.4 % but then remained stable from age 4 to 5 years. While verbalisations of certain foods being “healthy” were very uncommon at ages 3 and 4 (<1.0 %), a significant increase was found at age 5 when 8.9 % used the term “healthy”.

Explorations of whether children who displayed an awareness of body change strategies retained this awareness over time revealed that among just under half the children the understanding of food control as a body change strategy was stable over time. Specifically, for *diet awareness,* 45 % (*n* = 5) of the 11 children who demonstrated an understanding of this at age 3, continued to demonstrate an understanding at age 4. Furthermore, 47.5 % (*n* = 19) of the children who demonstrated an understanding at age 4, retained this at age 5. For *food awareness*, 44 % (*n* = 7) of the children who demonstrated an understanding of this at age 3, continued to demonstrate an understanding at age 4. Furthermore, 47 % (*n* = 22) of the children who demonstrated an understanding at age 4, retained this at age 5. In contrast, however, the understanding of physical exercise as a body change strategy was much less stable over time. Regarding *exercise awareness,* 0 % (*n* = 0) of the 6 children who demonstrated an understanding of this at age 3, continued to demonstrate an understanding at age 4. Furthermore, 20 % (*n* = 6) of the children who demonstrated an understanding at age 4, retained this at age 5. Regarding *physical activity awareness,* only 6 % (*n* = 1) of the 17 children who demonstrated an understanding of this at age 3, continued to demonstrate an understanding at age 4. Finally, 24 % (*n* = 10) of the 42 children who demonstrated an understanding at age 4, retained this at age 5.

In addition, we explored the co-occurrence of food-related and exercise-related responses. At age 3, of the 16 children who demonstrated a general *food awareness*, 0 % (*n* = 0) gave a response classed as *physical activity awareness* or *exercise awareness.* At age 4, of the 47 children who demonstrated *food awareness,* only 11 % (*n* = 5) gave a response demonstrating *physical activity awareness,* and 8.5 % (*n* = 4) provided a response pertaining to *exercise awarenes*s. Finally, at age 5, of the 76 children who demonstrated *food awareness,* 16 % (*n* = 12) gave a response demonstrating *physical activity awareness,* and 14.5 % (*n* = 11) provided a response related to *exercise awarenes*s.

### Gender and weight status differences

In a series of 2 × 2 chi-square analyses no significant gender differences were found on the four primary variables at Time 1, including diet awareness, *χ*^2^ (1) = 1.42, *p* = .35, food awareness, *χ*^2^ (1) = .07, *p* = 1.00, exercise awareness, *χ*^2^ (1) = 1.96, *p* = .23, and physical activity awareness, *χ*^2^ (1) = 3.32, *p* = .08. Gender differences were not found at any of the other time points either. At Time 1, Time 2, and Time 3 respectively, 27, 18 and 15 % of the children were overweight. Chi-square tests revealed no significant differences in responses according to weight status (*p* = .01).

## Discussion

The aim of the present study was to investigate the development of the awareness of dietary restriction and physical activity as body change strategies in very young girls and boys by prospectively examining the development of these understandings between the ages of 3 and 5 years old. Our findings suggest that children of these ages have varying conceptualizations of dietary restriction and exercising as means of body size change. Furthermore, it seems that awareness of such body change strategies have significantly increased by the age of 5, to the point that between 20 and 30 % of children mention the manipulation of food and physical activity to reduce body size by age 5.

A range of children verbalised that changes in eating behaviour could reduce body size and these verbalisations increased progressively between ages 3 and 5. Suggestions included reducing food intake or changing the quality of food eaten, which would be considered appropriate strategies by weight loss strategy advocates, including for children [31]. The concepts of “healthy” foods and “unhealthy” foods was rare at 3 and 4 years, but began to emerge in the 5-year-olds. The idea that some foods are healthier than others can potentially be useful for children to make sensible food choices; however, if it is also associated with some foods being “good” and others “bad”, it may lead to the development of food rules which have been shown to be associated with disordered eating and food preoccupations [32, 33]. Investigating the outcomes of children with early conceptions of “good” and “bad” foods will be important to further understand the role of these food rules in the development of later eating pathology.

More worryingly, some children also suggested stopping eating altogether. While this may reflect somewhat unsophisticated, dichotomous thinking styles in this age group [34], it is still an approach linked to fasting behaviour. It was also concerning that some 3 to 5 year olds made suggestions for weight loss behaviours that are associated with other disordered eating patterns. A few children, particularly boys, suggested vomiting food eaten and spitting out the food in order to change from a large to a small figure. Encouragingly, all but one of these children suggested purging at only one time point, which suggests it generally is temporary as the dominant idea. Future research should examine the extent to which young children who suggest fasting or purging develop extreme weight loss methods in later years and are at greater risk of developing disordered eating.

At the age of five, children generally could not define the word “diet” when asked, with only 8 % of children associating the word with food and about half of those noting its association with food rules. The fact that so few appeared to be familiar with the word is encouraging in relation to their developing dieting proneness. However, many more children did appear to understand the concept of dietary restriction as they described food-related weight loss behaviours, even if they did not recognise the word “diet”. Our findings that a very small minority of 5-year-olds understood the term “diet” although a larger proportion had an awareness of dietary restriction for weight loss are consistent with Lowe and Tiggemann’s (2003) findings in similarly aged Australian children. In contrast, a U.S. study indicated that a larger proportion of 5-year-old girls (up to 45 %) had some conception of dieting (Abramovitz & Birch, 2000), suggesting that there may be cultural differences in the development of this terminology, or the measurement of children’s awareness.

Nonetheless, the apparent lack of understanding of the word “diet” in most young children has implications for measurement of the construct in very young children. Other words, or descriptions, of related tendencies appear to be needed to assist children to understand what is being assessed. In our study, of the 10 children who correctly suggested that a “diet” involves food rules, three associated it with a parent’s diet. Therefore, parental modelling may have been involved for the minority of children who were aware of the definition of diet, consistent with findings that parent weight loss behaviours are associated with child attitudes and behaviours [35].

A subsample of children also referred to physical activity and exercise as methods for reducing body size, and, similarly to dietary restriction, awareness of these strategies increased with age. Children were able to name specific forms of exercise such as running or going to the gym, as well as mentioning more child-appropriate forms of physical activity such as playing outside. While physical activity has been shown to play a protective role against the development of paediatric obesity [3], it has only had limited success in obesity interventions for children [36] and similar patterns have been found among adults. In contrast, popular media, including TV shows and magazine content, suggest that physical exercise is one of the most effective means of weight loss [37, 38]. Thus, young children may be learning from the prevailing cultural discourse that exercise is an important strategy for weight loss. This is problematic, for a number of reasons. First, the belief that weight and shape are highly malleable through exercise can increase beliefs regarding the controllability of obesity and resulting stigmatisation of obese individuals [39, 40]. Second, these beliefs may lead to feelings of ineffectiveness when they are not helpful in obtaining sustainable weight-loss, which may result in the development of more extreme forms of weight control behaviours, including excessive exercise and disordered eating. Interestingly, responses suggesting an awareness of physical activity as a body change strategy did not necessarily co-occur with responses indicating an awareness of food-related strategies, suggesting that these might develop independently in children.

A subset of children made comments suggesting negative associations with large body sizes, and awareness of stigma associated with fat people. These include responses such as the larger figure child being naughty or being ostracised, and included one child making the extreme suggestion that the large figure should kill herself and start growing again. The estimated proportions of children expressing these ideas here should be treated with caution given that this study was not specifically designed to illicit such attitudes. Consistent with this possibility, other studies, for example, have demonstrated that only 16 % of children aged 4–6 years chose a chubby doll when asked which they were most likely to play with, out of a selection including average weight and thin dolls [39], suggesting that in fact, in this age group, the majority of children hold negative attitudes towards overweight individuals. Thus negative stereotyping and stigma related to overweight appears at this early age in some children, as also reported by other researchers [27].

As expected, and consistent with previous findings in 5-year olds, gender differences in proportion of children suggesting food-related or physical activity related answers were not found in this sample [15]. Therefore, there does not appear to be a differential awareness of particular weight loss strategies at this early age, although at later ages female adolescents have been found to more often favour use of dieting strategies and boys exercise strategies to change body size and shape [41]. Therefore, these gender differences in the use of dietary restraint compared to exercise as body change strategies in adolescents may not be related to gender differences in the *awareness* of such strategies, but rather the way in which these strategies are related to gendered body *ideals* with females more likely to desire thinness and males to desire muscularity. It would be important to explore gender differences in beliefs regarding appropriate weight control methods as children continue to age.

The present study has a number of limitations. First, the proportions of children who verbalised each theme may be an underestimate of the proportion that would endorse those themes if children had been directly asked about their views of various eating and exercise strategies. However, the approach used here, of allowing children to generate their own suggestions for responses to body size changes and methods for reducing body size, taps into the most *salient* conceptualisations, and can minimise suggestibility effects that occur in young children, and avoid children responding to closed questions they do not fully understand [42, 43]. Furthermore, it ensured that no unhealthy weight control strategies were taught to the children as a result of the interview. In addition, while our analyses combined the responses to the two open-ended questions, the wording of the two questions differed resulting in the first question perhaps being more likely to elicit beliefs, and the second awareness per se. Other limitations include the presence of direct assessment of understanding of the term ‘diet’ at only one time point; however, the fact that so few children could define the word even at 5 years old, suggests that familiarity with the term develops mostly at a later time in this cultural context.

Nevertheless, the current study is the first to track very young children from 3 to 5 years old, to ascertain their understanding of body size changes, and perceptions of whether eating behaviour and exercise can influence body size. The qualitative data can provide a resource for further development of tools for assessing these constructs in young children. Furthermore, exploring the ways in which the early development of the awareness of dietary restriction and exercise as means of body size change predict the development of later unhealthy eating and exercise behaviours may help inform prevention efforts and improve the identification of individuals at high risk for these concerns. Interventions aiming to prevent paediatric obesity and body image and eating concerns, and promote the use of healthy weight control strategies throughout the lifespan, should consider that some attitudes may already be present in very young children.

## Conclusions

The awareness of dietary restriction and physical exercise as strategies for weight loss and body change emerges as young as 3-years-old, and significantly increases from 3- to 5-years-old. Such awareness is important as it might lay the ground for future behaviours that constitute important risk factors for eating disorders. Interventions aiming to promote healthy means of weight control and obesity prevention should consider that certain attitudes may already be present in very young children.
